# Supplementary material for: Back to the basics: Clinical assessment yields robust mortality prediction and increased feasibility in low resource settings
Source: PLOS Glob Public Health. 2023 Mar 29;3(3):e0001761. doi: 10.1371/journal.pgph.0001761 (PMC10057736; doi:10.1371/journal.pgph.0001761)
Supplement: S1 Table — (DOCX) [file pgph.0001761.s001.docx]

**S1 Table. Injury severity, mechanism, and disposition outcomes by sex.**

|  | | Percentage (n) | | p-value |
| --- | --- | --- | --- | --- |
|  | | Male | Female |  |
| HEAIS (Mean, n) | | 2.19 (6577) | 1.89 (2785) | <0.001* |
| Age (Median, n) | | 31.5 (6712) | 31.3 (2845) | 0.66 |
| Mechanism of Injury | |  |  |  |
|  | RTI | 57.1 (3808) | 54.6 (1547) | 0.029* |
|  | Assault | 13.4 (895) | 14.4 (408) | 0.195 |
|  | Fall | 12.1 (807) | 16.8 (477) | <0.001 |
|  | Stab/cut | 10.3 (690) | 4.4 (124) | <0.001* |
| Disposition | |  |  |  |
|  | Discharged home | 60.8 (3997) | 72.3 (2036) | <0.001* |
|  | Left against medical advice | 15.1 (992) | 10.1 (284) | <0.001* |
|  | Admitted ward | 13.4 (880) | 10.7 (300) | <0.001* |
|  | Transferred | 5.5 (362) | 3.5 (98) | <0.001* |
|  | Died | 2.57 (169) | 1.31 (37) | <0.001* |
|  | Directly to operating room | 2.1 (138) | 1.7 (49) | 0.255 |
|  | Admitted to intensive care unit | 0.55 (36) | 0.39 (11) | 0.324 |

HEAIS = highest estimated abbreviated injury scale; RTI = road traffic injury

* = statistically significant p-value (less than 0.05)
