# Supplementary material for: Back to the basics: Clinical assessment yields robust mortality prediction and increased feasibility in low resource settings
Source: PLOS Glob Public Health. 2023 Mar 29;3(3):e0001761. doi: 10.1371/journal.pgph.0001761 (PMC10057736; doi:10.1371/journal.pgph.0001761)
Supplement: S2 Table — (DOCX) [file pgph.0001761.s002.docx]

**S2 Table. Logistic regression of disposition outcomes.**

|  | **Discharged home**  **aOR (95%CI)** | **Left AMA** | **Admit ward** | **Transferred** | **Died** | **Directly to OR** | **Admitted to ICU** |
| --- | --- | --- | --- | --- | --- | --- | --- |
| Female Sex | 1.32**  (1.2-1.5) | 0.73**  (0.6-0.8) | 0.91  (0.8-1.1) | 0.86  (0.7-1.1) | 0.74  (0.5-1.2) | 1.11  (0.8-1.6) | 0.89  (0.4-1.8) |
| HEAIS | 0.12**  (0.1-0.13) | 1.90**  (1.8-2.0) | 1.98**  (1.9-2.1) | 3.05**  (2.8-3.4) | 10.2**  (8.1-13) | 2.53**  (2.2-2.9) | 2.67**  (2.1-3.3) |
| RTI | 0.65**  (0.5-0.8) | 2.67**  (2.0-3.6) | 1.34*  (1.0-1.7) | 0.48**  (0.3-0.7) | 1.01  (0.5-1.9) | 1.15  (0.6-2.1) | 0.54  (0.2-1.2) |
| Assault | 2.02**  (1.5-2.7) | 1.26  (0.9-1.8) | 0.64*  (0.5-0.9) | 0.23**  (0.1-0.4) | 1.31  (0.6-3.1) | 0.48  (0.2-1.2) | 0.10*  (0.01-0.8) |
| Fall | 1.12  (0.9-1.5) | 1.92**  (1.4-2.7) | 1.17  (0.9-1.6) | 0.37**  (0.2-0.6) | 0.53  (0.2-1.4) | 1.23  (0.6-2.5) | 0.32  (0.1-1.2) |
| Stab/cut | 4.54**  (3.3-6.3) | 0.78  (0.5-1.2) | 0.43*  (0.3-0.6) | 0.44**  (0.3-0.7) | 0.57  (0.2-1.5) | 1.79  (0.9-3.6) | 1  (1) |

aOR = adjusted odds ratio; 95%CI = 95% confidence interval; AMA = against medical advice; OR = operating room; ICU = intensive care unit; HEAIS = highest estimated abbreviated injury scale; RTI = road traffic injury

* = p-value less than 0.05

** = p-value less than 0.001
