# Supplementary material for: Back to the basics: Clinical assessment yields robust mortality prediction and increased feasibility in low resource settings
Source: PLOS Glob Public Health. 2023 Mar 29;3(3):e0001761. doi: 10.1371/journal.pgph.0001761 (PMC10057736; doi:10.1371/journal.pgph.0001761)
Supplement: Attachment — Submitted filename: Response to Reviewers.docx [file pgph.0001761.s004.docx]

Dear PLOS Global Public Health Editors and Reviewers,

Thank you very much for your comments and edits. Please review our responses below to each point raised in the editing process.

Sincerely,

Mark Yost

**Journal Requirements:**

1. Please send a completed 'Competing Interests' statement, including any COIs declared by your co-authors. If you have no competing interests to declare, please state "The authors have declared that no competing interests exist".

The authors have declared that no competing interests exist.

2. Please amend your detailed Financial Disclosure statement. This is published with the article. It must therefore be completed in full sentences and contain the exact wording you wish to be published.
   a. State the initials, alongside each funding source, of each author to receive each grant.

The Cameroon Trauma Registry was supported by University of California San Francisco and University of California Los Angeles Departments of Surgery research funding (Dr. Catherine Juillard) and NIH R21TW010453 grant (Dr. Catherine Juillard, Dr. Alain Chichom-Mefire, and Dr. S. Ariane Christie).

This publication was supported in part by the H H LEE RESEARCH PROGRAM grant (Dr. Mark T. Yost). H H LEE RESEARCH PROGRAM had no role in study design, data collection and analysis, decision to publish, or preparation of the manuscript.

3. In the online submission form, you indicated that "Data available on reasonable request". All PLOS journals now require all data underlying the findings described in their manuscript to be freely available to other researchers, either 1) In a public repository, 2) Within the manuscript itself, or 3) Uploaded as supplementary information. This policy applies to all data except where public deposition would breach compliance with the protocol approved by your research ethics board. If your data cannot be made publicly available for ethical or legal reasons (e.g., public availability would compromise patient privacy), please explain your reasons by return email and your exemption request will be escalated to the editor for approval. Your exemption request will be handled independently and will not hold up the peer review process but will need to be resolved should your manuscript be accepted for publication. One of the Editorial team will then be in touch if there are any issues.

We will upload our de-identified data as an excel file as “Supporting information.”

4. Please upload all main figures as separate Figure files in .tif or .eps format only. For more information about how to convert and format your figure files please see our guidelines.

Figure file saved in .tif format. Appropriate file will be reuploaded. Figure title and caption inserted into text of manuscript as per websited instructions.

5. Please review your reference list to ensure that it is complete and correct. If you have cited papers that have been retracted, please include the rationale for doing so in the manuscript text or remove these references and replace them with relevant current references. Any changes to the reference list should be mentioned in the rebuttal letter that accompanies your revised manuscript. If you need to cite a retracted article, indicate the article’s retracted status in the References list and also include a citation and full reference for the retraction notice.

References were reviewed, there appear to be no citations of papers that have been retracted. If you have any concerns regarding a particular article in the reference list, please do not hesitate to contact.

Correction applied to reference #19 (Hay et al., 2017), previous draft incorrectly indicated publishing journal as Institute for Health Metrics and Evaluation. Reference is now corrected to show that article was published in The Lancet.

Correction applied to reference #30 (Afuwape et al., 2011), first author mistakenly listed twice in reference.

**Review Comments to the Author**:

**Reviewer #1**: The authors report a well conducted study to estimate injury severity and predict mortality in resource-constrained settings more easily. The use of the physical-exam based Highest Estimated Abbreviated Injury Score (HEAIS) has great promise in triaging patients and managing hospital resources. While the study and results are very well presented, I have a few questions for the authors:

1. The results indicate that HEAIS is statistically inferior to KTS and RTS but with good overall discrimination of mortality. It is therefore concerning that the authors conclude in the discussion section that HEAIS is “comparable” to KTS and RTS. This may give the erroneous impression that HEAIS is equivalent to KTS and RTS in predicting mortality. Could the authors address this?

Thank you for your comment. You are correct that such a characterization is misleading. We have made the following changes to the Discussion section on pages 13-14 (lines 255-258): “When compared to scoring systems such as RTS and KTS, HEAIS has superior feasibility with slightly lower predictive capacity of mortality in a resource-limited setting. Though statistically inferior to KTS and RTS mortality predictions, the difference in mortality predictive capacity remains small and HEAIS demonstrates excellent overall discrimination of mortality.”

2. It would be good for the authors to clarify if patients with HEAIS scores had data for ALL anatomic locations? If this was not the case, an explanation of how this may or may not affect the results would be helpful

Thank you for your comment.

The following sentence was added to the Results section on page 11 (lines 193-194): “Among the population with calculated HEAIS, 86.8% (n=8361) had HEAIS data for all anatomic locations.”

The following sentences have been added to the Discussion section on page 14 (lines 273-280): “HEAIS without data for all anatomic locations would only affect results if the injury with the greatest severity was not logged in the CTR. Such an occurrence seems unlikely as it would constitute a major error in data collection among a regularly supervised, trained research team. Among those without HEAIS for all anatomic locations, it is much more likely that data omission for an anatomic location occurred because findings in the location were unremarkable.”

3. The authors clearly identified missing “Respiratory Rate” as main reason for not being able to calculate KTS or RTS. It would be good for the authors to comment in the discussion section on the possibility of HEAIS + RR in increasing injury severity predictiveness in resource-constrained settings.

Thank you for your comment. The HEAIS score does not rely on vital sign data; thus, increased collection of respiratory rates in the CTR would not impact the number of patients with HEAIS.

The following additions have been made to the Discussion section on page 15 (lines 300-303): “The improvement of respiratory rate data collection would allow for the calculation of KTS and RTS for thousands of CTR patients. While this improvement would increase the feasibility of KTS and RTS scores in the Cameroonian context, the scores remain less feasible than HEAIS.”

**Reviewer #2**:

1. Manuscript addresses the important public health problem in sub-Saharan Africa (SSA) of injury related death and disability. The tool is feasible and was tested in several hospitals with different characteristics and catchment areas.

Thank you for your comment.

2. The authors state that “HEAIS scores were completed at a greater rate for young patients and female patients compared to RTS and KTS”. This is not well described in the result section. It is described as ORs in table 4 but should be mentioned clearly in a sentence or two in the result section

Thank you for your comment. The following sentence was added to the Results section on page 11 (lines 196-197): “Female and younger patients were more likely to possess adequate data to calculate HEAIS rather than RTS or KTS (Table 4).”

3. In the methods section, hospitals are described as first, second, third and final facility, but described in the tables as site A, B, C, D. I suggest using the same description in both the method and the result section to decrease confusion.

Thank you for your comment. We intentionally labelled the hospitals in this manner to preserve institutional anonymity and ensure that the reader would not be able to discern which hospital had data missingness difficulties. Institution specific data are provided privately to promote improvement through a culture of feedback. We did not wish to foster a sense of distrust or comparison among our partner institutions by highlighting their shortcomings in a public manner.

4. The scale being tested required less use of technology, was more feasible and may alleviate data disparities in women as trauma patients. Are the authors able to include more details as to the types or setting of injury? Do women experience different patterns of injury or are more demanding scales not used for women because women get less quality services compared to men?

Thank you for your questions. Our findings show that women are more likely to have data missingness when calculating injury severity scores that require more data, such as RTS and KTS. However, it does not appear that women suffer worse outcomes compared to men. We have included two supplementary data tables (Supplemental Tables 1 and 2) that address these calculations.

The following sentence was added to the Methods section on page 7 (lines 136-138): “We used logistic regression to test for associations in disposition outcomes controlling for sex, injury severity (e.g., HEAIS), and injury mechanism.”

The following paragraph was added to the Results section on page 13 (lines 234-238): “Women presented with less severe injury compared to men (mean HEAIS 1.87 vs. 2.19, p<0.001). Women experienced more falls than men (16.8% vs. 12.1%, p<0.001), but men suffered more RTIs (57.1% vs. 54.6%, p<0.029) and stab wounds (10.3% vs. 4.4%, p<0.001) (Supplemental Table 1). Logistic regression revealed that women had greater odds of discharge home from the ED (Supplementary Table 2).

The following sentences was added to the Discussion section on page 14 (lines 262-265): “While women are more likely to demonstrate data missingness in the calculation of RTS and KTS, it does not appear that women suffer worse outcomes than men. Additional data would be needed to determine if greater odds of discharge home correlate with worse health.”

Upon review of our data, we noticed an error in table 4. The stab/cut mechanism of injury was found to be not significantly associated with HEAIS, RTS, KTS data missingness. We corrected the table and edited the Results section appropriately.

5. Description of local teams in each hospital as well as the local team of investigators is not very clear, were there local PIs at each site? I suggest acknowledging local teams who are not part of the authors.

Thank you for your comment. We will add the following sentence in the Acknowledgements section on page 17-18 (lines 352-355): “We acknowledge the dedication and effort of the Cameroonian trauma registry staff at each participating hospital who are involved in data entry and the support of the Cameroonian Ministry of Public Health.”
